# Development of a Self-Healing Gel with Self-Healing Kinetics That Can Be Controlled by Heat

**DOI:** 10.3390/gels10060410

**Published:** 2024-06-19

**Authors:** Rikuto Saito, Shingo Tamesue

**Affiliations:** Division of Engineering and Agriculture, Graduate School of Regional Development and Creativity, Utsunomiya University, Yoto 7-1-2, Utsunomiya 321-0904, Tochigi, Japan; rikuto0214saichann@docomo.ne.jp

**Keywords:** self-healing, stimuli-responsive materials, smart gels, soft matter, kinetics control

## Abstract

A self-healing gel with self-healing kinetics that can be regulated by heat is developed. The gel is composed of a polymer having benzophenone (BP) substituents, which are cross-linked with a main alkyl chain via ester bonds, titanium chloride, and zinc. This gel material shows a self-healing property at room temperature. Also, its self-healing behavior can be accelerated by heating the gel. This gel having self-healing kinetics that can be regulated by heat is favorable for practical use. When we want to use a self-healing property as a stop-gap measure, a rapid self-healing property is demanded. On the other hand, when we want materials repaired beautifully or decomposed surfaces need to be attached beautifully, a slow self-healing property is favorable. These opposite demands can be answered by the gel with self-healing kinetics that can be regulated by heat.

## 1. Introduction

Gel materials are composed of polymeric networks and solvents (water [1,2,3], organic solvents [4,5,6], or ionic liquids [7,8,9]) and have high elasticity and softness [10,11,12,13]. Because of these characteristics, gel materials are expected materials to be used for constructing soft robots [14,15], artificial muscles [16,17], adhesives [18,19,20,21], and others [22,23,24,25,26,27].

Among the gel materials, stimuli-responsive gel materials [28,29] are expected to be used for artificial actuators, stimuli-responsive smoke screens, and so on. Due to their usefulness, various stimuli-responsive materials have been enthusiastically studied and reported. For example, photo-responsive gel materials can be formed by using polymers having the side chains interacting via host–guest interactions [30]. The gel composed of a polymer having cyclodextrins and a polymer having azobenzene moieties as side chains shows photo-responsive sol-gel transitions, adhesion, and actuating motion.

Recently, gel materials having self-healing abilities were developed for creating materials having a long lifespan [31,32,33,34]. For example, self-healing gel materials using host–guest interaction between cyclodextrins and their guest compounds were reported [35].

It is possible to reduce consuming ingredients by using self-healing materials having long lifespan, and these materials will help us create an ecofriendly world.

When we design and develop self-healing materials, the kinetics control of self-healing by stimuli, such as photo and heat, is important. Materials for which self-healing kinetics can be controlled by stimuli will help us use their self-healing properties more conveniently. It is favorable to self-heal rapidly when the decomposed materials need to be healed rapidly. On the other hand, it is favorable to self-heal slowly when the decomposed materials need to be healed accurately.

In this research, a self-healing material for which self-healing kinetics can be controlled by heat using a polymer that has ester substituents as side chains and titanium chloride (TiCl_4_) and zinc was developed and reported. These self-healing materials help us use them by adjusting their self-healing kinetics to the objectives for which they are used.

## 2. Results and Discussion

### 2.1. Development and Fabrication of Self-Healing Gels

**Scheme 1:**
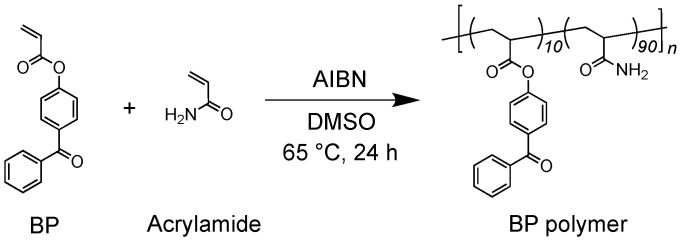
Synthesis of BP polymer composed of BP and acrylamide. The synthesized BP polymer was a random copolymer composed of 10 mol% of BP and 90 mol% of acrylamide.

First, a BP polymer, which has benzophenone substituents as side chains, was synthesized via the free radical polymerization of BP, for which synthetic detail is shown in the experimental section, and acrylamide with 2,2′-(diazene-1,2-diyl)bis(2-ethylpropaneitrile) (AIBN) as a radical initiator in dimethylsulfoxide (DMSO) as shown in Scheme 1 and as described in the Experimental section.

**Figure 1:**
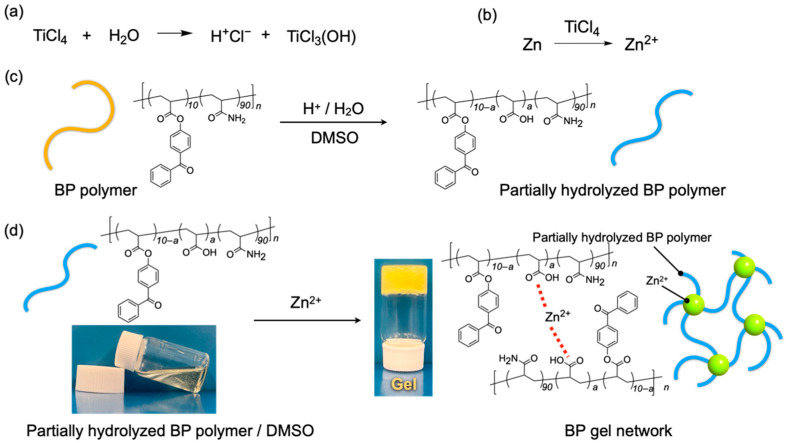
(**a**) Reaction of TiCl_4_ with H_2_O existing in the air and in dimethylsulfoxide (DMSO), and (**b**) formation of zinc ion (Zn^2+^) by the reaction of zinc and TiCl_4_. (**c**) Scheme of forming partially hydrolyzed BP polymer from BP polymer by the hydrolysis reaction with HCl formed in (**a**). (**d**) Photograph of formation of BP gel network by coordination bonds between partially hydrolyzed BP polymer and zinc ion (Zn^2+^) formed in (**b**). The gel was composed of 19 wt% of BP polymer, 0.5 M of TiCl_4_, and 1.0 M of zinc.

The synthesized BP polymer (0.3 g) was mixed with titanium chloride (TiCl_4_, 0.5 M) and zinc powder (1.0 M) in 1.0 mL of DMSO. Afterwards, it was confirmed that the mixture gelated as shown in Figure 1. In the mixture, TiCl_4_ and H_2_O reacted and released a proton (H^+^) as shown in Figure 1a. Simultaneously, zinc reacted with TiCl_4_ and formed a zinc cation (Zn^2+^) as shown in Figure 1b.

The ester linkages of the BP polymer were hydrolyzed partially by the protons formed in Figure 1a as shown in Figure 1c. The resultant partially hydrolyzed BP polymer having carboxylic acids was cross-linked by Zn^2+^ via a coordinate bond [36,37] as shown in Figure 1d. It was considered that the gelation was carried out by these reactions of the BP polymer, TiCl_4_, and zinc.

### 2.2. SEM Observation of Self-Healing Gels

**Figure 2:**
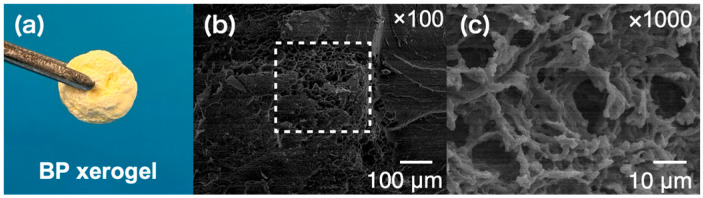
(**a**) Photograph of the xerogel prepared using the gel composed of BP polymer, TiCl_4_, and zinc. The original BP gel before exchanging the solvent from DMSO to water was composed of 19 wt% of BP polymer, 0.5 M of TiCl_4_ and 1.0 M of zinc. (**b**,**c**) Scanning electron microscope images of BP xerogel. The white rectangle dashed line in (**b**) shows the observation area of (**c**).

Scanning electron microscope (SEM) images were observed to evaluate the morphology of the gel as shown in Figure 2. To remove DMSO, the prepared gel was soaked into ion-exchanged water for 24 h twice to exchange the solvent from DMSO to water. After that, the gel was lyophilized as shown in Figure 2a and used for SEM observation. The obtained SEM images showed that the BP polymer formed a network structure as shown in Figure 2b,c. It is considered that the BP polymers were cross-linked by metal ions and formed a stable gel network.

### 2.3. Self-Healing Ability of Gels

**Figure 3:**
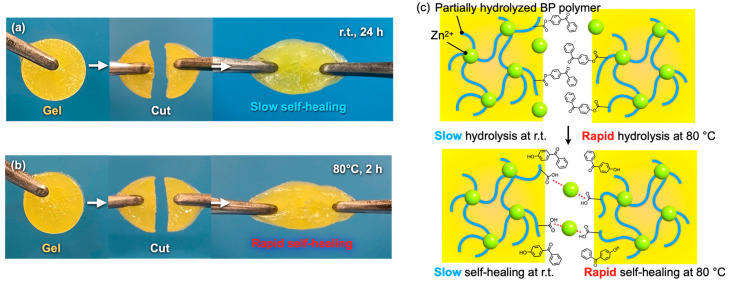
Photographs of the self-healing test of the gels composed of 19 wt% of BP polymer, 0.5 M of TiCl_4_, 1.0 M of zinc, and 1.0 mL of DMSO at (**a**) room temperature and at (**b**) 80 °C. (**c**) Schematic illustration of self-healing mechanism and difference of self-healing kinetics between room temperature and 80 °C.

The gel composed of 19 wt% of BP polymer, 0.5 M of TiCl_4_, and 1.0 M of zinc was cut into two pieces with a razor as shown in Figure 3. After that, the cut pieces were brought into contact with each other and were left to stand for 24 h at room temperature. Consequently, the cut pieces self-healed and formed an original gel again as shown in Figure 3a. The self-healed gel could be pulled with pairs of tweezers without separating again.

The gel pieces were heated at 80 °C in the same manner as at room temperature. The gel pieces were self-healed and formed one gel piece again more rapidly at 80 °C than at room temperature as shown in Figure 3b. It was confirmed that the cut gel pieces adhered again in 2 h. Considering that the gel self-healed after 24 h at room temperature, the gel self-healed much more rapidly at 80 °C. In this way, the self-healing could be accelerated by heating the cut gel pieces after they were brought into contact with each other. It is considered that the difference of the self-healing kinetics between room temperature and 80 °C was caused by the difference in the hydrolysis kinetics of the ester groups as shown in Figure 3c. The hydrolysis reaction of the ester groups linking between benzophenone and the polymer chain were more rapid at 80 °C than at room temperature and made the gel networks cross-linked again rapidly.

### 2.4. Self-Healing Test of Reference Samples

**Figure 4:**
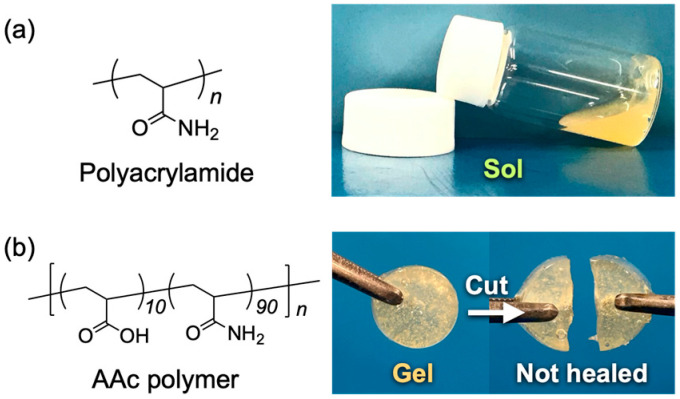
(**a**) Chemical structure of polyacrylamide and photograph of a DMSO solution of 19 wt% of polyacrylamide mixed with TiCl_4_ (0.5 mM) and zinc powder (1.0 M). (**b**) Chemical structure of AAc polymer and photograph of a DMSO gel formed by mixing 19 wt% of AAc polymer with TiCl_4_ (0.5 M) and zinc powder (1.0 M). The resultant gel did not have self-healing ability like BP gel, and the cut gel pieces were separated easily by pulling them even after 48 h.

To evaluate the self-healing mechanism of the gel, some reference samples were prepared as shown in Figure 4. Firstly, the polyacrylamide was used for preparing a sample instead of the BP polymer. The prepared sample did not gelate as shown in Figure 4a. It was considered that the acrylamide polymer not having ester groups did not form carboxylic acid groups, which interact with zinc ions even after mixing TiCl_4_ and zinc. Hence, the sample did not form a gel network. Second, a sample was composed of a polymer synthesized from acrylic acid and acrylamide (AAc polymer), TiCl_4_, and zinc. The polymer did not contain ester groups in its chemical structure and had a structure in which the BP substituents were removed from the structure of the BP polymer. After mixing all the components, the solution gelated. However, the gel did not have a self-healing ability as shown in Figure 4b. It is supposed that the gel composed of the AAc polymer, TiCl_4_, and zinc did not self-heal because the AAc polymers had too many carboxylic acid groups, which could interact with zinc ions, and there were few free zinc cations, which could interact with the carboxylic acid of the AAc polymers and be used for self-healing in the gel. From the result, it is considered that the degree of the hydrolysis of ester linkages affects the self-healing ability and mechanical strength critically.

### 2.5. Indentation Test

**Figure 5:**
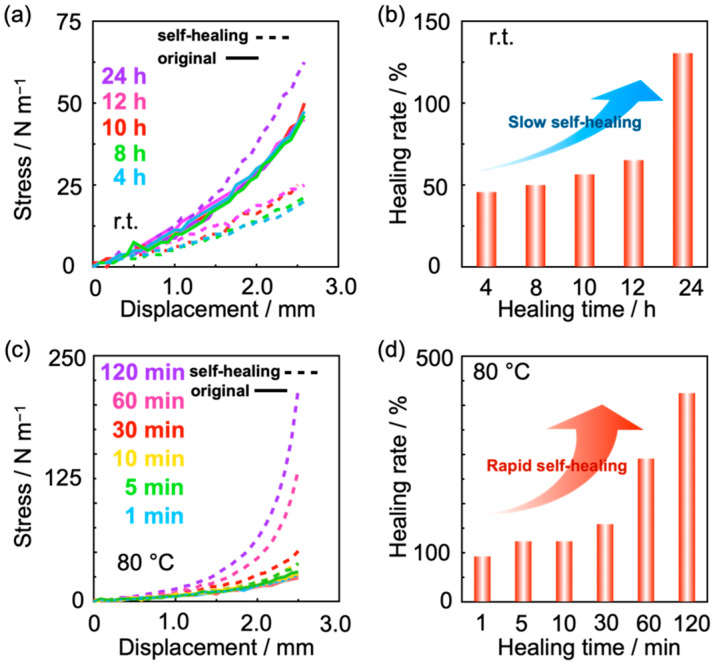
(**a**) Displacement and stress curves of the DMSO gel composed of BP polymer, TiCl_4_, and zinc before and after self-healing at room temperature. The rigid lines show the results of the original BP gels, and the dash lines show those of the self-healed gels. (**b**) Self-healing rate depending on the self-healing time at room temperature calculated from (**a**). Self-healing rate was the value that the inclination of the curve of self-healed interface divided with that of the original gel. (**c**) Displacement and stress curves of the DMSO gel composed of BP polymer, TiCl_4_, and zinc before and after self-healing at 80 °C. (**d**) Self-healing rate depending on the self-healing time at 80 °C calculated from (**c**). The bold lines and the dash lines in (**a**,**c**) showed the results of the original gels, and those of the self-healed gels, respectively. The gels used for the experiments were composed of 19 wt% of BP polymer, 0.5 M of TiCl_4_, and 1.0 M of zinc.

An indentation test was carried out to evaluate the mechanical strength of the self-healed interfaces as shown in Figure 5. Figure 5a shows the dependency of the mechanical strength of the self-healed interfaces on the self-healing time at room temperature. As can be seen, the mechanical strength increased as the self-healing time increased. The concentration dependency of the self-healing rates calculated from the obtained results of Figure 5a was shown in Figure 5b. The self-healing rates were calculated from the inclination of displacement-stress curves of the original gels and the self-healed gels. The self-healing rates of the self-healed interfaces increased as the concentrations of the carboxylic acid in the gel increased.

Time-course changes of the mechanical strength of the self-healing interfaces were measured at 80 °C. As can be seen in Figure 5c,d, the mechanical strength of the self-healing interfaces recovered rapidly at 80 °C compared with room temperature. In this way, the results of the indentation test indicated that the interfaces self-healed rapidly by heating. After 5 min of self-healing time, the self-healing efficiency was over 100% because the hydrolysis of ester groups progressed, and the resultant carboxylic acids interacted with zinc ions more than before the self-healing experiment. It is supposed that the difference in the self-healing kinetics is due to the difference of the hydrolysis reaction progress at room temperature and 80 °C.

### 2.6. ^1^H NMR Spectroscopy

**Figure 6:**
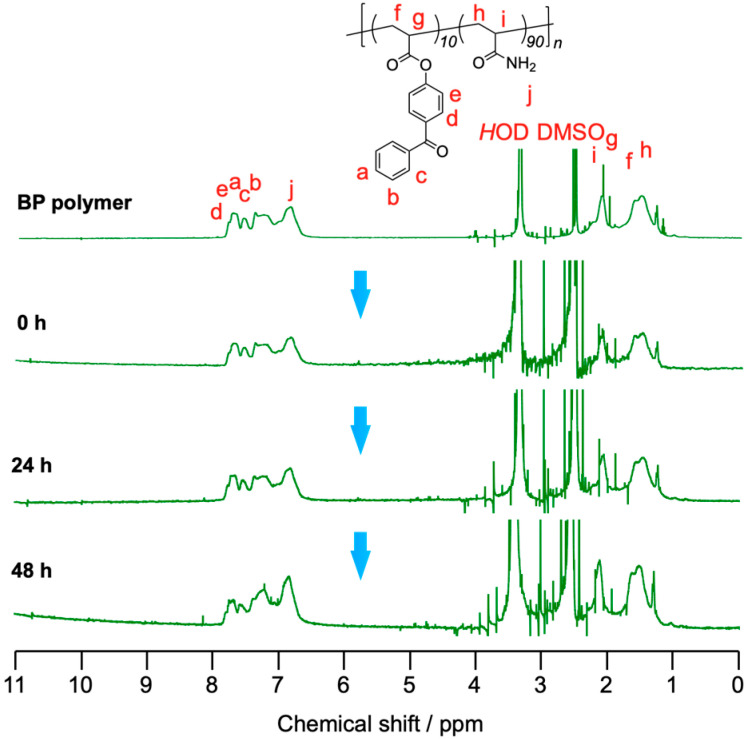
Time-course change of ^1^H NMR spectra (500 MHz, DMSO-*d*_6_, r.t.) of BP polymer (19 wt%) mixed with TiCl_4_ (0.5 M) and zinc powder (1.0 M) in DMSO at room temperature.

**Figure 7:**
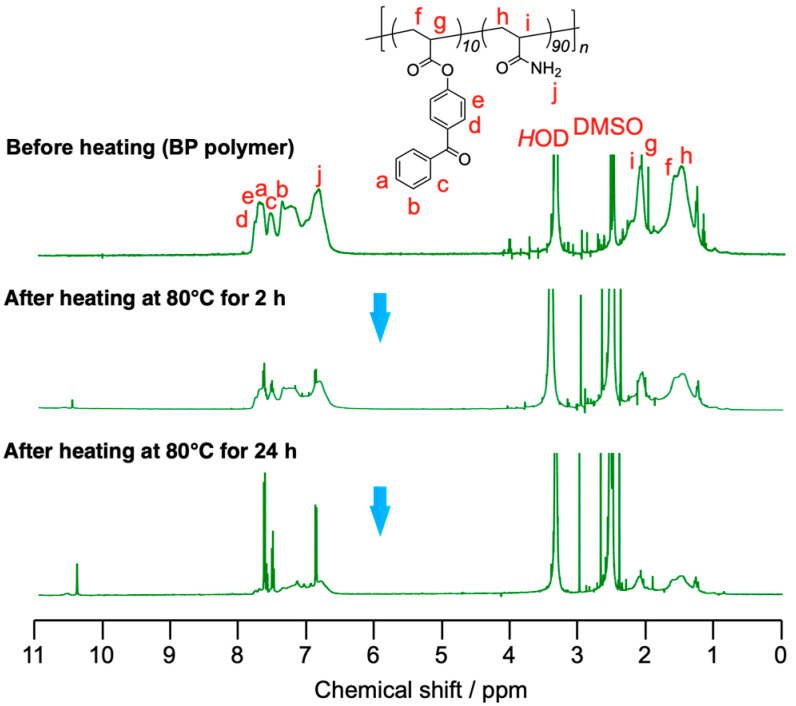
Time-course change of ^1^H NMR spectra (500 MHz, DMSO-d6, r.t.) of 19 wt% of BP polymer mixed with TiCl4 (0.5 M) and zinc powder (1.0 M) in DMSO and heated at 80 °C.

^1^H NMR spectroscopy was carried out to evaluate the mechanism of the self-healing phenomenon of the gel composed of the BP polymer, TiCl_4_, and zinc as shown in Figure 6 and Figure 7. Figure 6 showed the result of ^1^H NMR spectroscopy at room temperature. The pattern of the signals assigned to the protons of the aromatic groups of the BP polymer around 6.6–7.8 ppm changed as time passed because the ester groups were decomposed by TiCl_4_. In particular, the signal intensity at 6.9 ppm increased because 4-hydroxyacetophenone formed by the hydrolysis with TiCl_4_. The formed multiple carboxylic acid interacted with zinc cations, and then the polymers were cross-linked via the coordination bonds between formed carboxylic acids and zinc cations. Figure 7 showed the result of ^1^H NMR spectroscopy at 80 °C. As can be seen in the figure, the hydrolysis reaction was accelerated by heating at 80 °C. The signals assigned to 4-hydroxybenzophenone were increased much faster and were seen more sharply and clearly than at room temperature. The difference in changes of the NMR spectra based on temperature indicates that the self-healing kinetics can be regulated by heat.

Next, the hydrolysis of the BP polymer was evaluated by using a model compound, 4-acetoxybenzophenone, as shown in Figure S5. Proton signals of 4-hydroxybenzophenone appeared in the ^1^H NMR spectra of 4-acetoxybenzophenone in deuterated DMSO by 48 h after the BP polymer with TiCl_4_ and zinc at room temperature and at 80 °C. Stronger proton signals of hydrolyzed 4-hydroxybenzophenone were observed from the sample heated at 80 °C, and it was confirmed that the kinetics of the hydrolysis differed depending on the temperature. These changes also support the hypothesis that the BP polymer formed a gel and showed the self-healing ability due to the hydrolysis of ester linkages.

### 2.7. Fourier-Transform Attenuated Total Reflection Infrared (FT-ATR-IR) Spectroscopy

**Figure 8:**
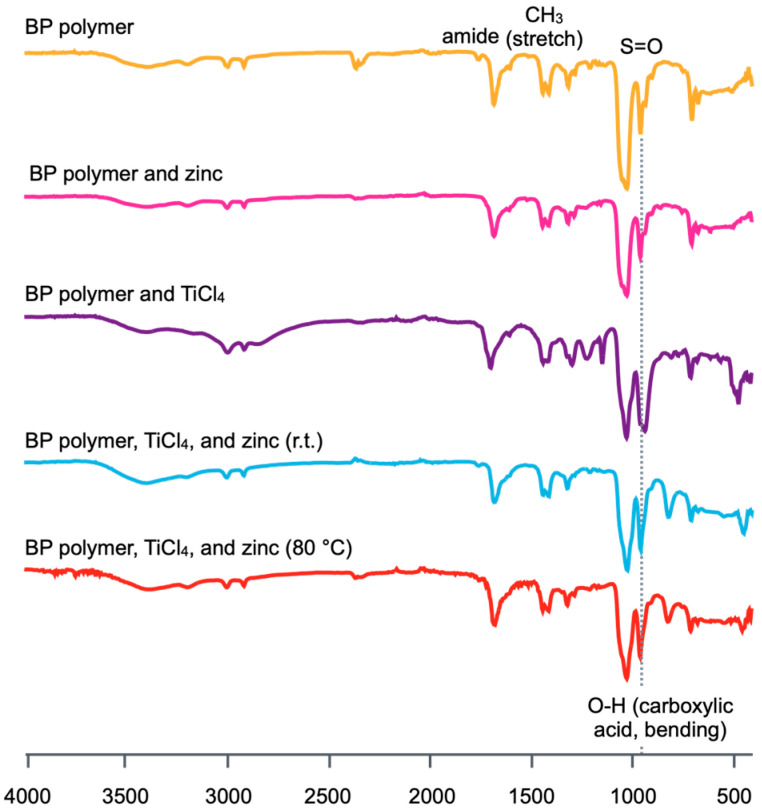
FT-ATR-IR spectra of the samples: BP polymer (orange), BP polymer mixed with zinc (pink), BP polymer mixed with TiCl_4_ (purple), and BP polymer mixed with TiCl_4_ and zinc at room temperature (light blue) and at 80 °C (red).

Fourier-transform attenuated total reflection infrared (FT-ATR-IR) spectroscopy was carried out to evaluate the mechanism of the gelation and the self-healing as shown in Figure 8. As can be seen in the figure, there is no significant spectral change in the spectrum of the sample composed of the BP polymer and zinc compared with that of the BP polymer. On the other hand, it was confirmed that the absorption band at 900 cm^−1^ assigned to the out-on-plane bending motion of the carboxylic acid groups increased in the spectra of the samples mixed with TiCl_4_. This spectral change means that the carboxylic groups in the BP polymer decomposed to carboxylic acid by the reaction with TiCl_4_. In this way, the results obtained from IR spectroscopy support the hypothesis that the gel formation and self-healing property of the gels composed of BP polymer, TiCl_4_, and zinc were due to the hydrolysis of ester groups by TiCl_4_.

## 3. Conclusions

Controlling self-healing kinetics is important when self-healing materials are intended for practical use. Slow self-healing is favorable when we make materials self-heal accurately so as not to leave any scars on the surfaces. On the other hand, fast self-healing is favorable when we make materials self-heal quickly as a stopgap measure. In this research, we developed a self-healing material with self-healing kinetics that can be regulated by temperature. In this self-healing system, ester bonds cross-linking between a polymer main chain and benzophenone substituents were hydrolyzed depending on temperature and formed carboxyl acid groups at the main chains. These carboxy acid groups interacted with zinc ions and bridged polymer chains via coordinal bonds. It is expected that the self-healing materials with controllable kinetics reported in this manuscript would help us construct a sustainable and eco-friendly society using self-healing materials having long lifespan because the self-healing kinetics can be changed depending on objectives for which they are used. Moreover, it is expected that it will become possible to make artificial skins having more superior self-healing abilities than the skin of our human bodies that cannot control the kinetics of self-healing. This can be done by continuing the studies of the self-healing materials for which self-healing kinetics are controllable like the gel reported in this research.

## 4. Materials and Methods

### 4.1. Materials and Measurement Apparatus

Acrylamide (AAm), triethylamine, acetone, ethylacetate, tetrahydrofuran (THF), 2,2′-(diazene-1,2-diyl)bis(2-ethylpropaneitrile) (AIBN), hydrochloric acid (HCl), sodium chloride (NaCl), magnesium sulfate (anhydrous), dimethylsulfoxide (DMSO), DMSO-*d*_6_, and chloroform-*d*_1_ were purchased from Kanto Chemical Co. Inc., Tokyo, Japan. The 4-hydroxybenzophenone, acryloyl chloride, and zinc (powder) were purchased from Tokyo Chemical Industry (TCI), Tokyo, Japan. All other reagents were purchased from Kanto Chemical Co. Inc., Tokyo, Japan. ^1^H NMR and ^13^C NMR spectra were recorded on a Varian model 500-MR spectrometer, Los Angeles, CA, USA. The indentation test was performed using Imada MX2-500N-FA, Toyohashi, Aichi, Japan, equipped with Imada ZTA-50N, Toyohashi, Aichi, Japan. The rate of extending samples was 10 mm/min. Infrared (IR) spectroscopy was conducted using a JASCO FT/IR-4100, Tokyo, Japan. Scanning electron microscopy (SEM) images were recorded using JEOL JCM-5610LV scanning electron microscope, Tokyo, Japan. SEM samples were spin-coated with platinum using an ion sputter coater (Hitachi E-1030 Ion Sputter Coater), Tokyo, Japan.

### 4.2. Synthesis of 4-Acryloylbenzophenone (BP)

4-acryloylbenzophenone (BP) was synthesized according to the literature previously reported [38,39]. The 4-hydroxybenzophenone (25 mmol) and triethylamine (36 mmol) were dissolved into 25 mL of dried tetrahydrofuran (THF) in a 100 mL round bottom flask. The solution was cooled in an ice bath. Then, 25 mL of dried THF solution, in which acryloylchloride (50 mmol) was dissolved, was added to the solution dropwise, and the solution was stirred for 30 min. After that, the solution was filtrated, and the filtrate was collected. The solution was mixed with ethylacetate and water and extracted. The collected organic layer was washed with 0.1 M hydrogen chloride (HCl) aqueous solution and brine three times each. After magnesium sulfoxide was added to the solution, the solution was filtrated, and its solvent was evaporated. The compound was estimated as BP by ^1^H NMR spectroscopy as shown in Figure S1. The yield of the reaction was 84%.

### 4.3. Synthesis of BP Polymer

BP (10 mmol), AAm (90 mmol), and AIBN (12 mmol) were dissolved into 75 mL of DMSO in a 200 mL round bottom flask. After N_2_ gas was purged into the flask, the solution was heated at 65 °C for 10 h. Consequently, a clear viscous solution was obtained. After acetone was added to the solution, white precipitation took place. This precipitation was collected by filtration and washed with acetone several times. The resultant precipitation was dried in vacuo and collected as BP polymer. The compound was estimated by ^1^H NMR spectroscopy as shown in Figure S2.

### 4.4. Synthesis of Polyacrylamide

AAm (90 mmol) and AIBN (12 mmol) were dissolved into 75 mL of DMSO in a 200 mL round bottom flask. After N_2_ gas was purged into the flask, the solution was heated at 65 °C for 10 h. After acetone was added to the solution, white precipitation took place. This precipitation was collected by filtration and washed with acetone several times. The resultant precipitation was dried in vacuo and collected as polyacrylamide (pAAm). The compound was estimated by ^1^H NMR spectroscopy as shown in Figure S3.

### 4.5. Synthesis of Poly(acrylic acid-co-acrylamide) (AAc Polymer)

Acrylic acid (AAc, 10 mmol), AAm (90 mmol), and 2,2′-azobisisobutyronitrile (AIBN, 12 mmol) were dissolved into 75 mL of DMSO in a 200 mL round bottom flask. After N_2_ gas was purged into the flask, the solution was heated at 65 °C for 10 h. Consequently, a clear viscous solution was obtained. After acetone was added to the solution, white precipitation took place. This precipitation was collected by filtration and washed with acetone several times. The resultant precipitation was dried in vacuo and collected as poly(acrylic acid-co-acrylamide) (AAc polymer). The compound was estimated by ^1^H NMR spectroscopy as shown in Figure S4.

### 4.6. Synthesis of 4-Acetoxybenzophenone

The 4-acetoxybenzophenone used for evaluating the hydrolysis of the BP polymer was synthesized according to the literature previously reported [40].

### 4.7. Mixing TiCl_4_ and Zinc in DMSO

Zinc powder (10 mmol) was dispersed in 10 mL of DMSO and stirred at 0 °C for 10 min under N_2_ atmosphere. Titanium chloride (TiCl_4,_ 5.0 mmol) was added to the solution dropwise and heated at 70 °C for 2 h. The resultant mixture was used for preparing the gel after being cooled to room temperature.

### 4.8. Preparation of BP Gel

BP polymer (0.3 g) and the prepared 1.0 mL of the DMSO mixture of TiCl_4_ and zinc were mixed in a sample tube and heated at 80 °C for 24 h. In the resultant mixture, 1.0 M of zinc and 0.5 M of TiCl_4_ existed. After heating, the mixture formed a gel (BP gel), for which the solvent was DMSO.

## Data Availability

The original contributions of the research are included in the article. Further inquiries can be directed to the corresponding author.

## Supplementary Materials

The following supporting information can be downloaded at: https://www.mdpi.com/article/10.3390/gels10060410/s1, Figure S1: ^1^H NMR (500 MHz, CDCl_3_, r.t.) spectrum of 4-acryloylbenzophenone (BP); Figure S2: ^1^H NMR (500 MHz, DMSO-*d*_6_, r.t.) spectrum of BP polymer (10:90 (mol:mol)); Figure S3: ^1^H NMR (500 MHz, DMSO-*d*_6_, r.t.) spectrum of polyacrylamide (pAAm); Figure S4: ^1^H NMR (500 MHz, DMSO-*d*_6_, r.t.) spectrum of AAc polyner (10:90 (mol:mol)); Figure S5: ^1^H NMR spectra (500 MHz, DMSO-*d*_6_, r.t.) of (a) 4-acetoxybenzophenone before mixing with TiCl_4_ and zinc, and 4-acetoxybenzophenone left to stand for 48 h at (b) room temperature and (c) 80 °C after mixed with 0.5 M of TiCl_4_ and 1.0 M of zinc.
