# Supplementary material for: Development of a Self-Healing Gel with Self-Healing Kinetics That Can Be Controlled by Heat
Source: Gels. 2024 Jun 19;10(6):410. doi: 10.3390/gels10060410 (PMC11202672; doi:10.3390/gels10060410)
Supplement: Supplementary file 1 [file gels-10-00410-s001.zip › gels-3024988-supplementary.pdf]

Development of a self-healing gel which self-healing kinetics can be controlled by heat.

Rikuto Saito,† Shingo Tamesue†*

†Division of Engineering and Agriculture, Graduate School of Regional Development and Creativity, Utsunomiya University.

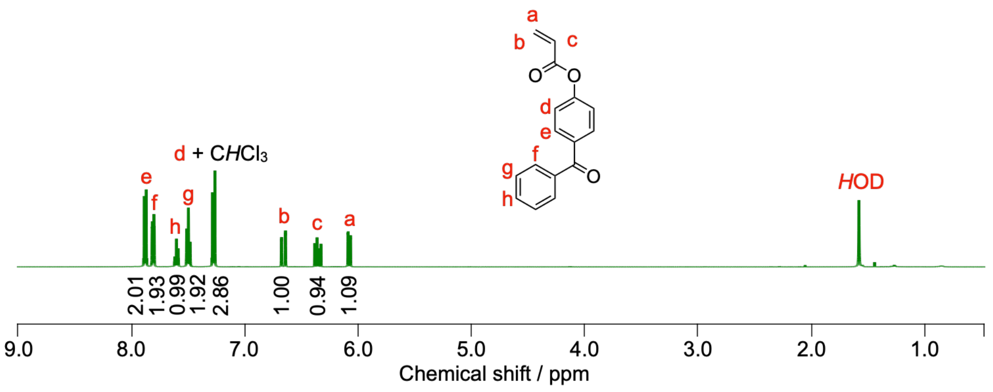

Figure S1. ^{1}H NMR (500 MHz, $CDCl_3$, r.t.) spectrum of 4-acryloylbenzophenone (BP).

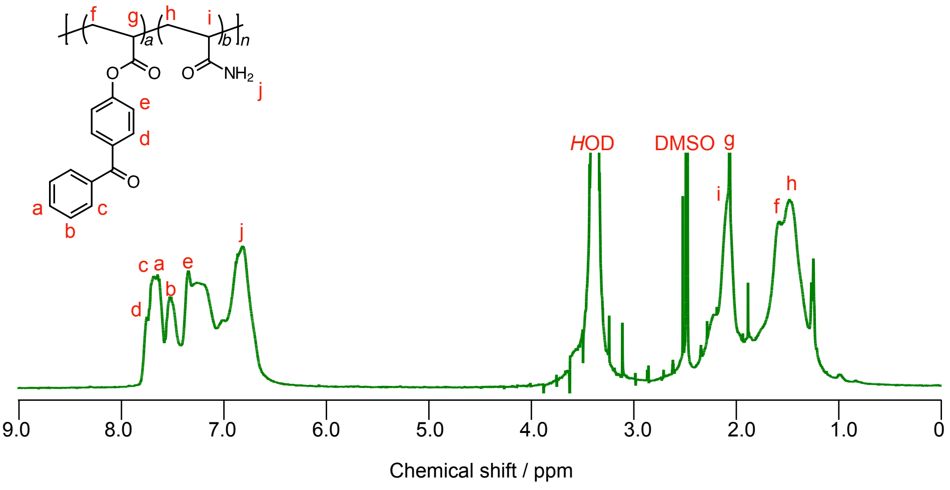

Figure S2. ^{1}H NMR (500 MHz, DMSO-d_6, r.t.) spectrum of BP polymer (10 : 90 (mol:mol)).

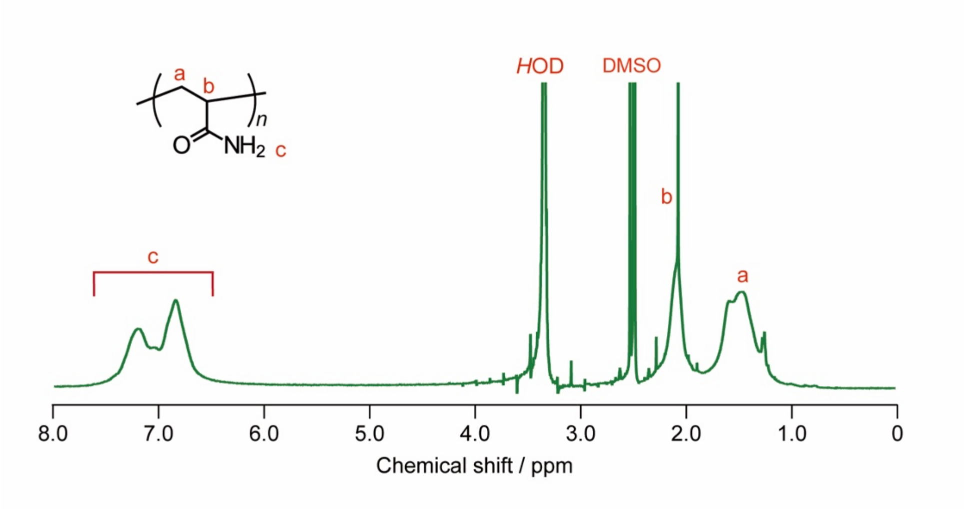

Figure S3. ^{1}H NMR (500 MHz, DMSO-d_6, r.t.) spectrum of polyacrylamide (pAAm).

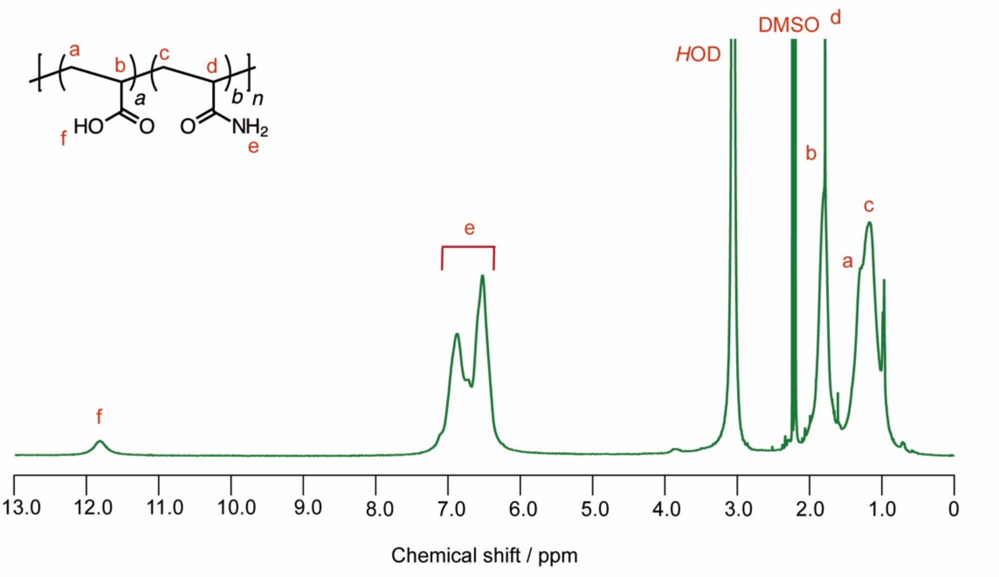

Figure S4. ^{1}H NMR (500 MHz, DMSO-d_6, r.t.) spectrum of AAc polyner (10 : 90 (mol : mol)).

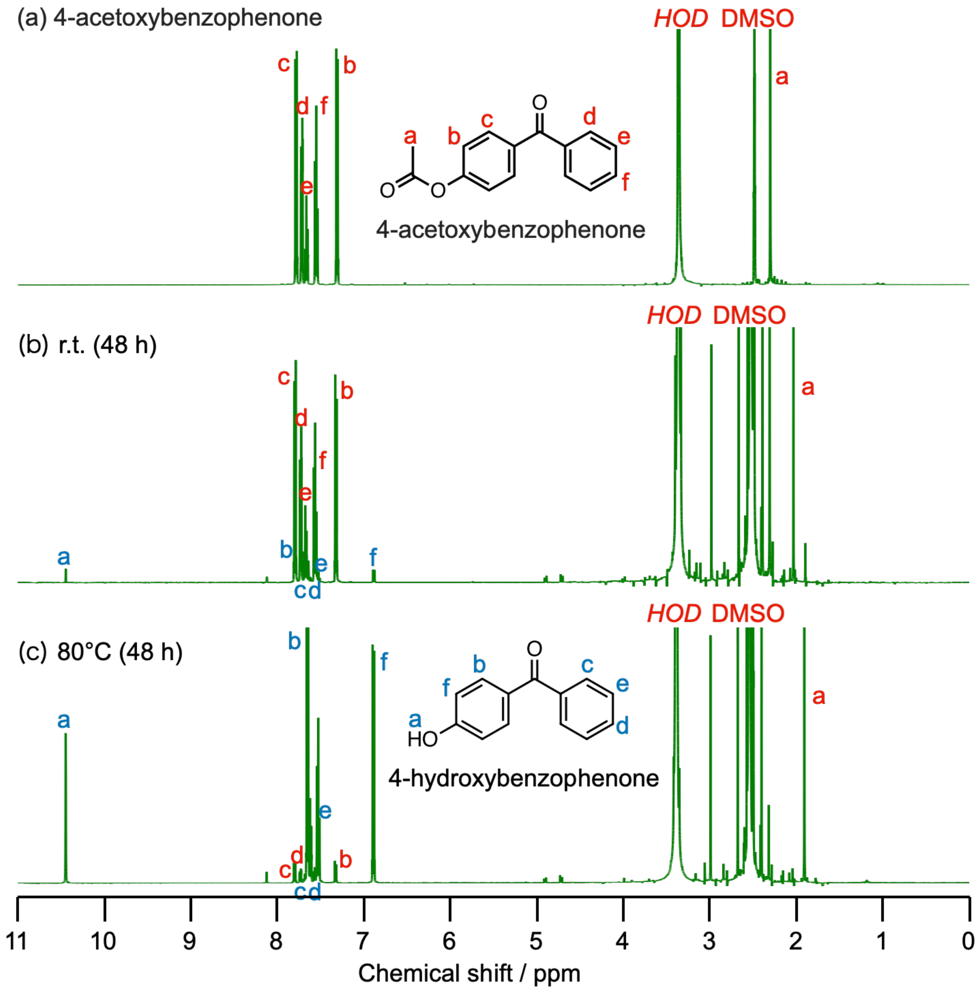

Figure S5. ^{1}H NMR spectra (500 MHz, DMSO-d_6, r.t.) of (a) 4-acetoxybenzophenone before mixing with $TiCl_4$ and zinc, and 4-acetoxybenzophenone left to stand for 48 hours at (b) room temperature and (c) 80 °C after mixed with 0.5 M of $TiCl_4$ and 1.0 M of zinc.
